# Relationship between both cardiorespiratory and muscular fitness and health-related quality of life in children and adolescents: a systematic review and meta-analysis of observational studies

**DOI:** 10.1186/s12955-021-01766-0

**Published:** 2021-04-21

**Authors:** Alberto Bermejo-Cantarero, Celia Álvarez-Bueno, Vicente Martínez-Vizcaino, Andrés Redondo-Tébar, Diana P. Pozuelo-Carrascosa, Mairena Sánchez-López

**Affiliations:** 1 Universidad de Castilla-La Mancha, Health and Social Research Center, Cuenca, Castilla-La Mancha, Spain; 2 Universidad Autónoma de Chile, Facultad de Ciencias de la Salud, Talca, Chile; 3 Universidad de Castilla-La Mancha, School of Education, Ciudad Real, Castilla-La Mancha, Spain; 4 Universidad Politécnica y Artística del Paraguay, Asunción, Paraguay

**Keywords:** Health-related quality of life, HRQoL, Physical fitness, Strength, Physical well-being, Psychological well-being, Children, Adolescents, Meta-analysis

## Abstract

**Background:**

No review to date has evaluated the association between physical fitness and health-related quality of life (HRQoL) in healthy children and adolescents. The aims of this systematic review and meta-analysis were to examine the relationship between both cardiorespiratory fitness (CRF) and muscular fitness (MF) and HRQoL in healthy subjects under 18 years of age and to describe the dimensions of HRQoL in which these relationships are more robust.

**Methods:**

The Medline, Embase, Cochrane Library, SCIELO, SPORTDiscus and PEDro databases were systematically searched to collect observational studies that examined the relationship between CRF and HRQoL and between MF and HRQoL in participants under 18 years of age without any diagnosed medical condition. Pooled effect sizes (ES) were estimated for the associations between both CRF and MF and the various HRQoL dimensions.

**Results:**

The pooled ES (95% CI) estimates for the relationship between CRF and HRQoL were as follows: 0.19 (0.10 to 0.27) for physical well-being, 0.19 (0.07 to 0.32) for psychological well-being, 0.20 (− 0.14 to 0.55) for perceived health status, 0.10 (0.00 to 0.20) for self-perception/self-esteem, 0.07 (− 0.05 to 0.19) for quality of family relationship, 0.14 (0.04 to 0.25) for quality of peer relationship, 0.17 (0.04 to 0.29) for everyday functioning at school and 0.20 (0.12 to 0.28) for total HRQoL score. The pooled ES (95% CI) estimates for the relationship between MF and HRQoL were: 0.25 (0.12 to 0.37) for physical well-being, 0.11 (0.04 to 0.17) for psychological well-being, 0.08 (0.01 to 0.15) for quality of family relationship, 0.14 (0.03 to 0.25) for quality of peer relationship, and 0.09 (0.03 to 0.14) for total HRQoL score.

**Conclusions:**

Our data suggest that both CRF and MF are positively associated with HRQoL, mainly in physical, psychological and peer relationships. Moreover, CRF is positively associated with school dimensions and MF is positively associated with family relationships.

*Trail registration* Protocol PROSPERO registration number: CRD42015025823.

**Supplementary Information:**

The online version contains supplementary material available at 10.1186/s12955-021-01766-0.

## Background

Quality of life (QoL) has been defined as the individual’s perception of their position in life in the context of the culture and value systems in which they live and in relation to their goals, expectations, standards and concerns [1]. Health-related quality of life (HRQoL) encompasses the aspects of overall quality of life that can be clearly shown to affect physical or mental health status [2]. The term refers to the effects of health, disease and treatments on QoL and excludes aspects that are not related to health, such as cultural, political or social conditions [3].

In children and adolescents, HRQoL includes the dimensions generally connected with daily activities, cognitive acquisitions, emotions, self-perception and interpersonal relationships, and the environment around them. In generic HRQoL questionnaires for children and adolescents, the dimensions most commonly measured are self-esteem, body image and autonomy, physical functioning or well-being, emotional status, family and social relationships, and school and leisure [4]. It is known that in children with poor HRQoL, normal development is impaired, making them less likely to mature into healthy adults [5].

Health-related physical fitness in youth is defined as a person's ability to perform physical activity (PA) and/or exercise, as well as attributes and capabilities that are associated with a low risk of developing chronic diseases and premature death [6]. The main health-related fitness components are cardiorespiratory fitness (capacity of the cardiovascular and respiratory systems to supply oxygen during sustained PA), musculoskeletal fitness (which includes muscle strength as the ability of the muscle to generate force, and flexibility as the ability of the muscle to move freely through a full range of motion) and motor fitness (which includes speed, agility and balance) [6]. Several studies have shown that health-related physical fitness, particularly cardiorespiratory fitness (CRF) and muscular fitness (MF), are associated with numerous physical, emotional, mental, and social health benefits in youth [7–9].

A recent systematic review [10] found that healthy children and adolescents who participated in higher levels of PA (any bodily movement produced by skeletal muscles that requires energy expenditure) had better HRQOL and, conversely, longer sedentary time was related to decreased HRQoL. It is important to note that although PA and fitness are closely related, as PA can improve MF and CRF, these are different concepts. On the other hand, the relationship between physical fitness and HRQoL has mainly been analyzed in the general adult population [11, 12]. It has also been studied in children and adolescents with health conditions such as epilepsy [13], diabetes [14], obesity [15] and asthma [16]. It has been suggested that in healthy children and adolescents, physical fitness [17–19] has a direct association with HRQoL, positively affecting aspects such as physical and psychological well-being, the relationship with peers or the school environment. Some authors [19, 20] have suggested there are differences between boys and girls in how certain attributes of physical fitness affect HRQoL.

It is unclear which HRQoL domains are more influenced by fitness level and whether different fitness components affect distinct domains. The main type of PA performed by children and adolescents could condition the improvement of certain specific fitness components. This fact, and the results of previous studies [19–21], may lead us to think that CRF and MF are differently associated with some HRQoL domains. However, and despite the fact that fitness levels at early ages (and the potential influence on HRQoL) tend to be maintained throughout life [22], no systematic review or meta-analysis has synthesized these relationships in healthy children and adolescents.

The purpose of this systematic review and meta-analysis was therefore to examine the relationship between physical fitness (CRF & MF) and HRQoL in healthy children (under 12 years old) and adolescents (12–14 years old and 15–17 years old) and to describe the HRQoL dimensions in which these relationships are more robust.

## Methods

### Protocol and registration

This systematic review was conducted in accordance with the PRISMA (Preferred Reporting Items for Systematic Reviews and Meta-Analyses) Statement [23] and the Cochrane Collaboration Handbook [24]. The protocol for this systematic review and meta-analysis has been registered in PROSPERO (registration number: CRD42015025823) and published elsewhere [25].

### Search strategy

The following electronic bibliographic databases were searched from their inception to Feb 2021 to identify relevant studies: Medline (via PUBMED), Embase, Cochrane Library, SCIELO, SPORTDiscus and PEDro. The search strategy combined the following keywords: “fitness”, “physical fitness”, “cardiorespiratory fitness”, “cardiovascular fitness”, “aerobic capacity”, “maximal oxygen uptake”, “peak oxygen uptake”, “VO2max”, “muscular strength”, “muscular endurance”, and “strength” with the terms “health-related quality of life”, “HRQoL”, “well-being”, “positive health”, “psychological health” and with “children”, “adolescent”, “young children”, and “schoolboy” (Table 1). References were imported into Endnote (Thompson Reuters, California, USA). Also, reference lists of relevant studies and previous systematic reviews and meta-analyses were identified to review the list of included studies.

**Table 1:** Sample search string for PubMed MEDLINE

Limits: publication languages: English, Spanish	
Truncation symbol: * = all possible word endings included	
**Query translation**	
("fitness"[All Fields]) OR "physical fitness"[All Fields]) OR "cardiorespiratory fitness"[All Fields]) OR "cardiovascular fitness"[All Fields]) OR "aerobic capacity"[All Fields]) OR "maximal oxygen uptake"[All Fields]) OR "peak oxygen uptake"[All Fields]) OR "VO2max"[All Fields]) OR "muscular strength"[All Fields]) OR "muscular endurance"[All Fields]) OR "strength"[All Fields]) AND (((("health-related quality of life"[All Fields] OR "HRQoL"[All Fields]) OR "well-being"[All Fields]) OR "positive health"[All Fields]) OR "psychological health"[All Fields])) AND ((("children"[All Fields] OR "adolescent"[All Fields]) OR "young children"[All Fields]) OR "schoolboy"[All Fields])AND (English[lang] OR Spanish[lang])	

### Eligibility criteria

Studies that examined the relationship between physical fitness and HRQoL in the general population of healthy children and adolescents were considered. The inclusion criteria were: (1) Participants: under 18 years of age, and without any diagnosed medical conditions, including obesity, diabetes, cancer or other chronic diseases; (2) Measurements: *HRQoL* conceptualized as a multidimensional construct composed of several domains which include physical, psychological, emotional and social aspects, measured by validated questionnaires with an acceptable internal consistency (Cronbach’s alpha > 0.7), either self-reported or reported by parents; *Cardiorespiratory fitness (CRF) and muscular fitness (MF)* measured by either laboratory tests, field tests or self-reported scales with demonstrated validity and reliability in children and adolescents. (3) Study design: Observational studies (cross-sectional, longitudinal). (4) Published in English or Spanish. No date limits were imposed in the search.

To make the review more focused and concrete, studies were excluded when they assessed specific constructs as outcomes, such as: (i) ‘stress’, ‘self-esteem’, ‘body image’, ‘anxiety’, ‘happiness’ ‘well-being’ or ‘depression’, which were not integrated within a validated multidimensional questionnaire to measure HRQoL because they do not measure that aspect of QoL in relation to health, i.e. they were not created to measure stress, anxiety, well-being or self-image according to the health status of the person, which is the aim of HRQoL questionnaires; (ii) ‘well-being” was also excluded when it was measured as a synonym for QoL, because this term refers to ‘‘a conscious cognitive judgment of satisfaction with one’s life’’, and HRQoL provides information on the way health affects well-being or QoL. Studies that reported their results with an overall fitness index without separating it into its components, or that measured sport participation or other components of fitness, such as speed, agility, balance, were also excluded, as they are closely related to the construct of motor competence (or motor skills) [26], making it difficult to determine when studies assessed motor competence or motor fitness, or flexibility, for which the evidence for health in children and adolescents is not as consistent as it is in the case of CRF and MF [6, 27, 28].

### Study selection

Two authors (ABC and MSL) independently screened the titles and abstracts of the studies identified in the search. Then, the full text of potentially eligible studies was re-evaluated. Where necessary, a third author (CAB) read the entire article to resolve any discrepancies.

### Data collection process

Two researchers (ABC and MSL) extracted the following data from the included studies using an ad hoc table (Table 2): study design, year, country, number of participants, age, instruments measuring CRF, MF and HRQoL results, and quality score. Disagreements regarding data extraction were resolved by consensus with a third author (CAB).

**Table 2:** Characteristic of the cross-sectional studies included in the review

Study	Country	n	Age (years)	HRQoL instrument	Fitness	Results
Andersen et al. [38]	Norway	1129	10	Kidscreen-27	CRF: Andersen test	↑CRF = ↑ HRQoL (↑ physical wellbeing, ↑ psychological well-being, ↑ autonomy and parents, ↑ social support and peers, ↑ school environment)
					MF: Handgrip test and the long jump test	↑Explosive strength in the lower body = Better autonomy and parents score
						No relationship between handgrip strength and HRQoL
Borras et al. [39]	Spain	302	11–12	CHIP-CE/PRF	CRF: 20 m shuttle run test	↑ CRF = ↑ physical well-being
Eddolls et al. [44]	United Kingdom	576	11–13	PedsQL 4.0	CRF: 20 m shuttle run test	↑CRF = ↑ physical well-being and HRQoL (total score mediated by physical well-being)
Evaristo et al. [40]	Portugal	567	12–18	Kidscreen-10	CRF: 20 m shuttle run test	↑ CRF = ↑ HRQoL
					MF: Handgrip test and the long jump test	↑ MF = ↑ HRQoL
Gálvez et al. [41]	Spain	298	8–12	Kidscreen-10	CRF: 20 m shuttle run test	↑CRF = ↑ HRQoL (Boys and girls)
Gerber et al. [21]	Switzerland	378	6–8	KINDL-R	CRF: 20 m shuttle run test	↑CRF = ↑physical wellbeing and ↑peer relationships
Marques et al. [17]	Portugal	3554	13–18	Kidscreen-10	CRF and MF: International Fitness Scale (IFIS)	↑CRF = ↑HRQoL, ↑self-rated health and ↑life-satisfaction (Boys and girls)
						↑MF = ↑HRQoL, ↑self-rated health and ↑life satisfaction (Boys and girls)
Morales et al. [19]	Spain	1.158	8–11	Kidscreen-52/Kidscreen-10	CRF: 20 m shuttle run test	Boys:
					MF: Handgrip test and the long jump test	↑CRF = ↑ physical well-being and Social support and peers
						↑MF = ↑ physical well-being, ↑Social support and peers, ↑social acceptance
						Girls: ↑CRF = ↑ physical well-being, ↑self-perception, ↑Social support and peers, ↑social acceptance, and ↑ HRQoL (Kidscreen-10 Index)
						↑MF = ↑ HRQoL and ↑physical well-being
Padilla-Moledo et al. [43]	Spain	684	6–16.9	HBSC	CRF: 20 m shuttle run test	↑CRF = ↑ life satisfaction and ↑ perceived health status
Palou et al. [18]	Spain	302	10–12	CHIP-CE/PRF	CRF: 20 m shuttle run test	↑CRF = ↑ physical well-being
Redondo-Tébar et al. [20]	Spain	1413	4–7	KINDL-R PRF	CRF: 20 m shuttle run test	Total sample:
					MF: Long jump test	↑CRF = ↑HRQoL (total score), ↑physical well-being, and ↑school
						↑MF = ↑HRQoL (total score), ↑physical well-being, and ↑school
						Boys:
						↑CRF = ↑physical well-being and ↑school
						↑MF = ↑physical well-being
						Girls:
						↑CRF = ↑HRQoL (total score), ↑school, ↑physical well-being and friends
						↑MF = ↑school
Saavedra et al. [42]	Spain	351	8–9	EQ-5D-Y, VAS	CRF: 20 m shuttle run test	↑CRF = ↑ perceived health status

*CRF* cardiorespiratory fitness, *MF* muscular fitness, ↑ increases or improves, *CHIP-CE* Child Health and Illness Profile, *EQ-5D-Y* EuroQol Group 5-dimension questionnaire, *HBSC* Health Behavior in School-aged Children, *HRQoL* Heath Related Quality of Life, *PRF* Parents report form, *VAS* Visual Analogue Scale

### Quality assessment

Two authors (ABC and MSL) independently assessed the methodological quality of the included studies. A third author (CAB) was consulted to resolve disagreements when necessary. The final decision on each assessment was agreed by all three authors. The methodological quality of the included studies was scored using a quality assessment list based on the Strengthening the Reporting of Observational studies in Epidemiology (STROBE) [29] and the Effective Public Health Practice Project (EPHPP) [30] criteria, which has been used in previous reviews [31, 32]. The list contains information on five categories: adequate description of the study sample (number of participants, mean age and sex); adequate assessment/reporting of HRQoL (measurement of the HRQoL construct and its domains by means of a validated questionnaire); adequate assessment of the fitness components (validity/reliability of the outcome measure reported and/or measurement procedure adequately described); adequate adjustment of confounders (at least sex and age); description of both the numbers and reasons for withdrawals and dropouts (participation rate at baseline at least 70%). For each study, the items on the list were rated as 1 or 0 depending on whether they met the criteria or not. A total quality score for each study was calculated by counting the number of positive items. The risk of bias of the studies was classified as high (0–2 positive items), medium (3 positive items) or low (4–5 positive items).

### Data synthesis and statistical analysis

The HRQoL dimensions included in the various questionnaires and which shared meaning were grouped into domains for analysis (Table 3). Effect size (ES) and 95% confidence intervals (CIs) for the association between children’s physical fitness (distinguishing between CRF and MF) and HRQoL were calculated for each report using Cohen’s d index [33]. We calculated the pooled ES for the total HRQoL score and each HRQoL dimension using a random-effects model based on the DerSimonian and Laird method [33]. When studies provided a linear regression b coefficient, it was used to calculate a standardized mean difference score [34, 35]. When it was not possible to estimate the pooled ES, a graphical representation of the individual ES was drawn.

**Table 3:** Grouping of variables by domains that share meaning

Domain	Denomination in the questionnaires
Physical well-being	Physical well-being [18–21, 38]
	Physical comfort [39]
	Physical QoL [44]
Psychological well-being	Psychological well-being [19, 21, 38]
	Emotional well-being [18, 20]
	Emotional comfort [39]
Quality of family relationship	Family [20, 21]
	Autonomy and parents [38]
	Quality of family relationship [43]
	Parents relationship [19]
Quality of peer relationship	Friends [20, 21]
	Social support and peers [19, 38]
	Quality of peer relationship [43]
Everyday functioning at school	Everyday functioning at school [21]
	School [20]
	School environment [38]
	Academic performance [43]
Perceived health status	Perceived health status [42, 43]
	Satisfaction with health [18, 39]
Self-perception/self-esteem	Self-perception [19]
	Self-esteem [20, 21]

The I^2^ statistic was used to assess heterogeneity across studies, with the following thresholds: not important (0–30%), moderate (30–50%), substantial (50–75%) and considerable (75–100%) [36]; p values were also considered. A sensitivity analysis was conducted by removing studies from the analysis one by one to analyze the influence of each study on the overall results and to detect whether any particular study accounted for a considerable proportion of heterogeneity. Finally, the funnel plot and the Egger test were used to examine publication bias [37]. STATA 14 (StataCorp) software was used to combine data.

## Results

### Study selection

**Fig. 1:**
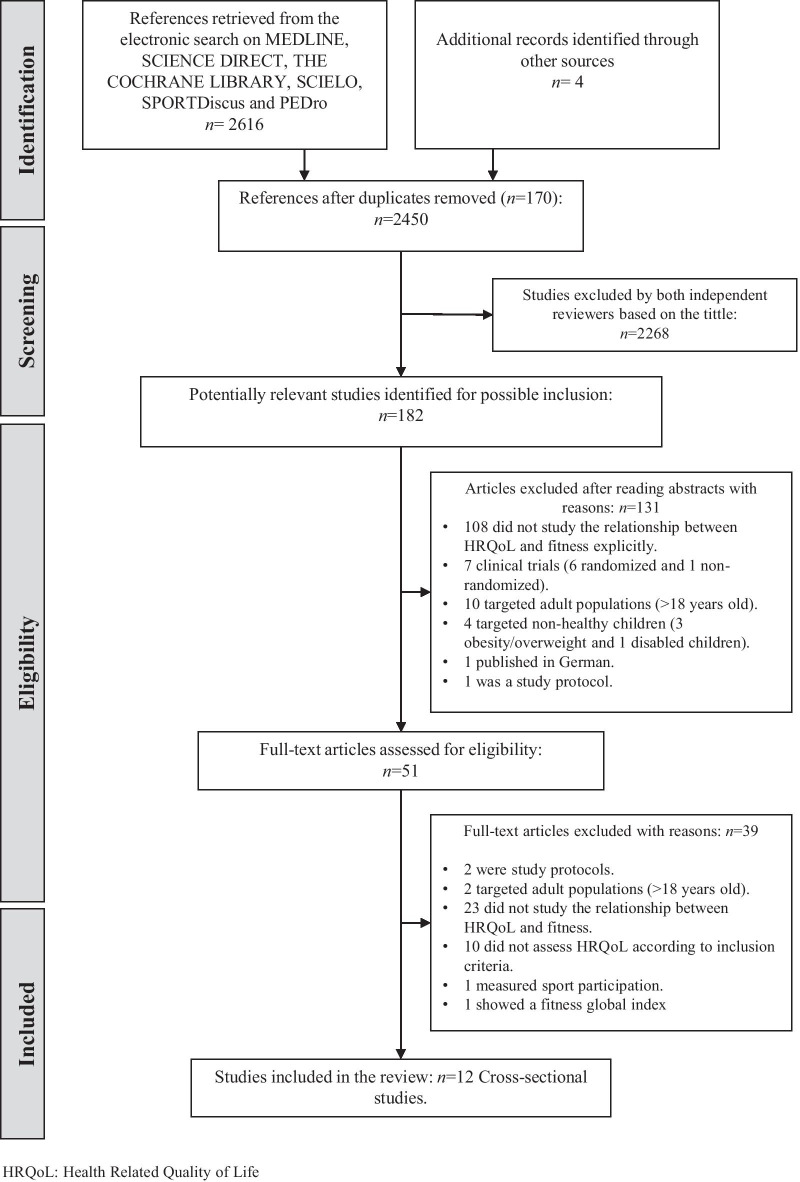
PRISMA Flow chart with the progress through the stages of study selection

This systematic search identified 2616 potentially relevant studies (Fig. 1). After removing 170 duplicates, 2268 studies were excluded based on the tittle. We screened 51 full texts in detail based on the title and abstract. Finally, a total of 12 studies [17–21, 38–44] met the inclusion criteria.

### Study characteristics and participants

The main characteristics of the included studies are shown in Table 2. The studies were published between 2011 and 2019 and all of them were cross-sectional. The total sample included 10,712 participants between 4 and 18 years of age without any health problems. The sample size ranged from 298 [41] to 3554 [17]. Only in one study [43] were the results separated for children (6–11.9 years) and adolescents (12–17.9 years). All studies involved both sexes but only four of them [17, 19, 20, 41] showed results by boys and girls separately. The studies included were from six different countries.

### Risk of bias

Table 4 shows the list of included studies with quality scores. Eight studies [17, 19–21, 38, 40, 42, 43] were rated as being at low risk of bias, two studies as medium risk [41, 44], and two studies as high risk [18, 39].

**Table 4:** List of included studies with quality scores

Study	Design	Assessment	1	2	3	4	5	Total score	Risk of bias
Andersen et al. [38]	Cs	CRF and MF	1	1	1	0	1	4	Low
Borras et al. [39]	Cs	CRF	1	0	1	0	0	2	High
Eddolls et al. [44]	Cs	CRF	1	0	1	1	0	3	Medium
Evaristo et al. [40]	Cs	CRF and MF	1	1	1	1	0	4	Low
Gálvez et al. [41]	Cs	CRF	1	1	1	0	0	3	Medium
Gerber et al. [21]	Cs	CRF	1	1	1	1	0	4	Low
Marques et al. [17]	Cs	CRF and MF	1	1	1	1	1	5	Low
Morales et al. [19]	Cs	CRF and MF	1	1	1	1	1	5	Low
Padilla-Moledo et al. [43]	Cs	CRF	1	1	1	1	1	5	Low
Palou et al. [18]	Cs	CRF	1	0	1	0	0	2	High
Redondo-Tébar et al. [20]	Cs	CRF and MF	1	1	1	1	1	5	Low
Saavedra et al. [42]	Cs	CRF	1	1	1	1	1	5	Low

*CRF* Cardiorespiratory fitness, *Cs* Cross-sectional, *MF* Muscular fitness

(1) Adequate description of the study sample (number of participants, mean age and sex); (2) Adequate assessment/reporting of HRQoL (measurement of the HRQoL construct and its domains by means of a validated questionnaire); (3) Adequate assessment of the physical fitness components (validity/reliability of the outcome measure reported and/or measurement procedure adequately described); (4) Adequate adjustment of confounders (at least sex and age); (5) Description of both the numbers and reasons for withdrawals and dropouts (participation rate at baseline at least 70%). “high risk = 0–2 score, “medium risk” = 3 score, and “low risk” = 4–5 score

### Measurements of HRQoL

Questionnaires used for measuring HRQoL varied across studies and included KINDL-R [20, 21], Kidscreen-52 [19], Kidscreen-27 [38], Kidscreen-10 [17, 19, 40, 41], EQ-5D with a Visual Analog Scale (VAS) [42], HBSC [43], PedsQL [44] and the CHIP-CE [18, 39]. The HRQoL questionnaires were self-administered in nine studies [17, 19, 21, 38, 40–44] and parent-reported in three studies [18, 20, 39].

### Cardiorespiratory fitness and health related quality of life

Ten studies [18–21, 39–44] assessed CRF using the 20-m shuttle run test [45], one study used the Andersen test [38], and one study used a self-reported scale, the International Fitness Scale (IFIS) [17]. Seven studies reported a positive relationship between CRF and total HRQoL score [17, 19–21, 40, 41, 44] and seven [18–21, 38, 39, 44] reported a positive relationship between CRF and physical well-being. In children and adolescents with higher CRF levels, four studies [19–21, 38] showed better peer relationship, three studies [17, 42, 43] shower better perceived health status, two studies shower better satisfaction with life [17, 43] and functioning at school [20, 38], one study [38] showed a positive relationship with psychological well-being and with quality of family relationship, and another [19] showed a positive relationship with self-perception in girls.

### Muscular fitness and health related quality of life

Five studies [17, 19, 20, 38, 40] reported the relationship between MF and HRQoL. MF was assessed using handgrip test (upper body isometric strength) [19, 38, 40] and the long jump test [19, 38] (lower body explosive strength). In one study [17], MF was measured using the IFIS scale. Morales et al. [19] and Evaristo et al. [40] used both strength tests to calculate an MF index (as the sum of the Z-scores of the two tests according to age and sex) to show their results. Andersen et al. [38] showed the results of handgrip strength and explosive strength in the lower body separately. Redondo-Tébar et al. [20] categorized MF into three quartiles (low, medium, and high).

All studies [17, 19, 20, 38, 40] reported a positive relationship between MF and total HRQoL score and only one study [38] reported no relationship between handgrip strength and HRQoL. The included studies showed positive relationships between MF and various HRQoL domains: physical well-being [19, 20], autonomy and parents [38], life satisfaction [17], social support and peers [19], self-rated health [17] and functioning at school (in girls) [20].

### Meta-analysis

**Fig. 2:**
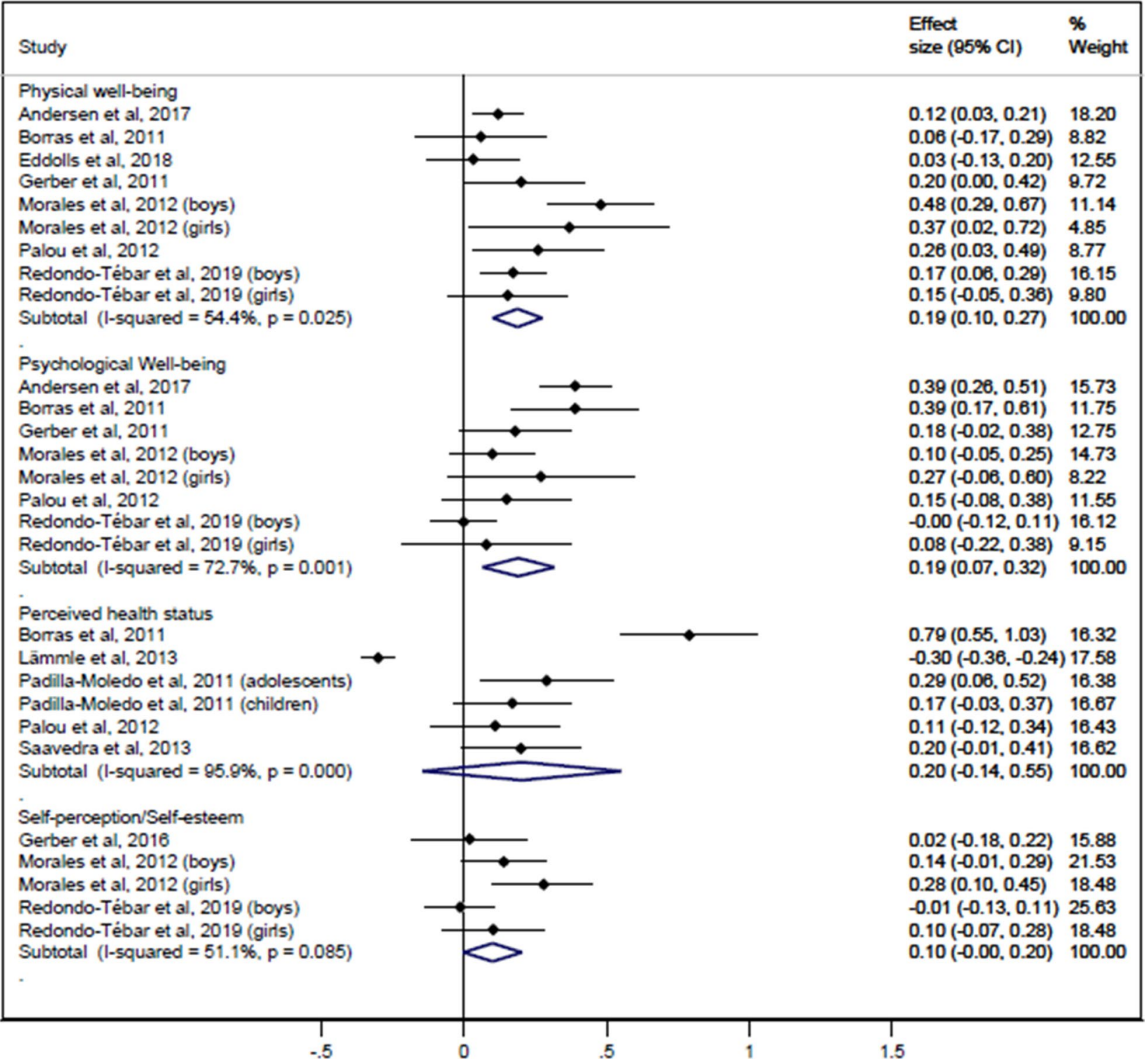
Pooled estimated effect size values of associations between CRF and physical well-being, psychological well-being, perceived health status, and self-perception/self-esteem

**Fig. 3:**
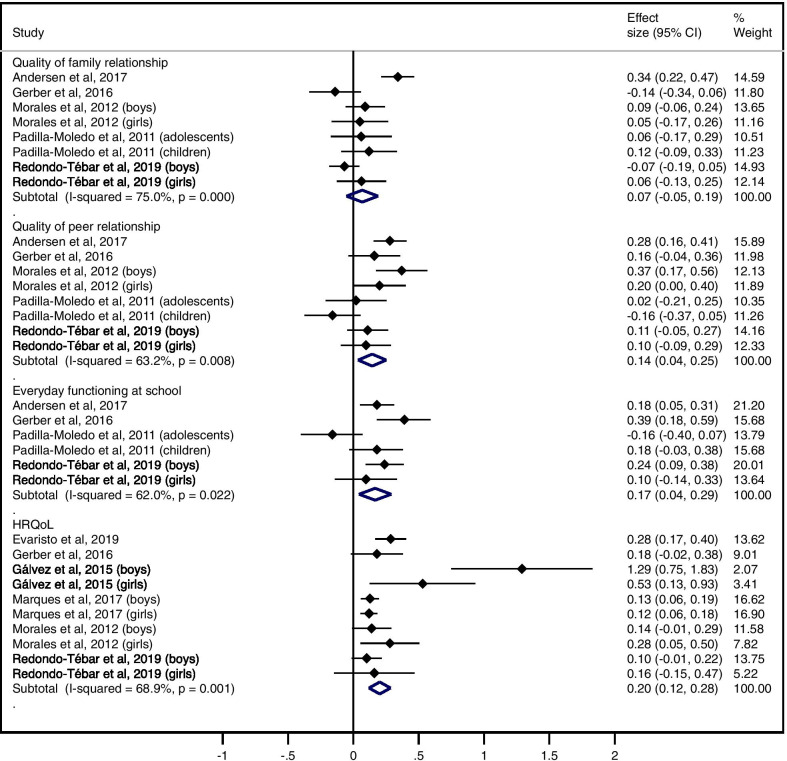
Pooled estimated effect size values of associations between CRF and quality of family relationship, quality of peer relationship, everyday functioning at school, and total HRQoL score

Figures 2 and 3 display the pooled ES (95% CI) estimates for the relationship between CRF and HRQoL: 0.19 (0.10 to 0.27) for physical well-being, 0.19 (0.07 to 0.32) for psychological well-being, 0.14 (0.04 to 0.25) for quality of peer relationships, 0.17 (0.04 to 0.29) for everyday functioning at school and 0.20 (0.12 to 0.28) for total HRQoL score. The pooled ES (95% CI) estimate was not statistically significant for self-perception/self-esteem: 0.10 (0.00 to 0.20), for perceived health status: 0.20 (− 0.14 to 0.55), or for quality of family relationship: 0.07 (− 0.05 to 0.19). Heterogeneity estimates ranged from substantial (I^2^ = 51.1% for Self-perception) to considerable (I^2^ = 95.9% for Perceived health status).

**Fig. 4:**
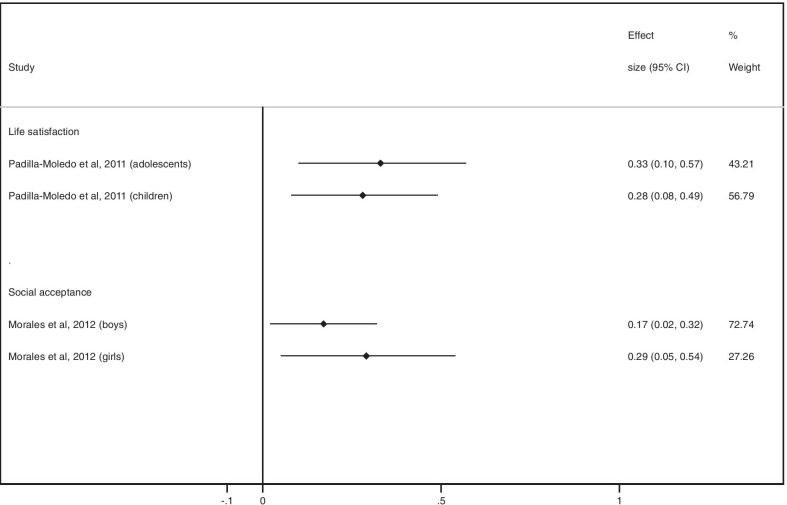
ES values of association between CRF and life satisfaction / social acceptance

ES (95% CI) for the relationship between CRF and life satisfaction was 0.28 (0.08 to 0.49) in children and 0.33 (0.10 to 0.57) in adolescents. ES for social acceptance ranged between 0.17 (0.02 to 0.32) in boys and 0.29 (0.05 to 0.54) in girls (Fig. 4).

**Fig. 5:**
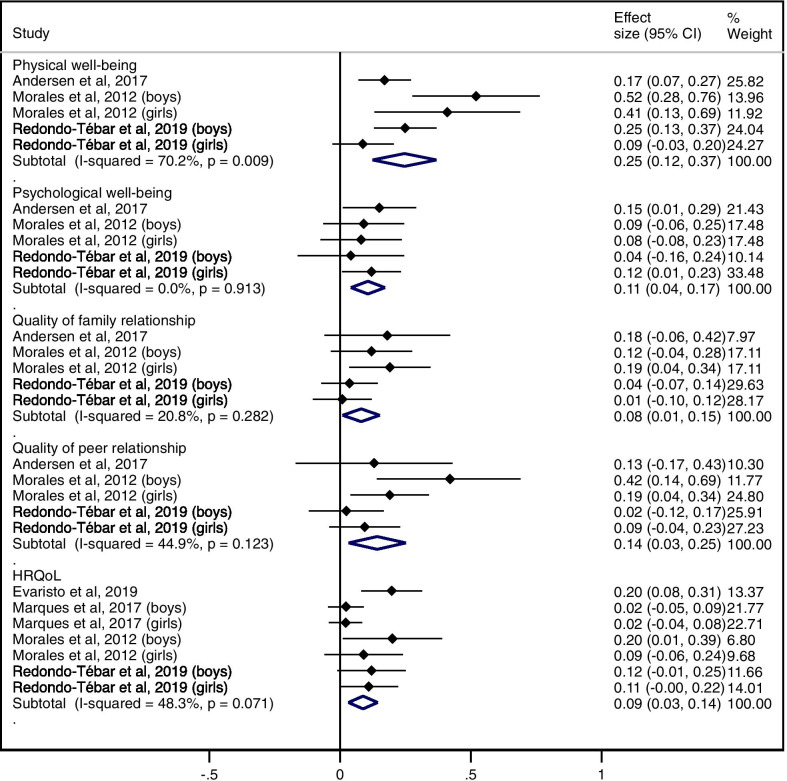
Pooled estimated effect size values of associations between MF and physical well-being, psychological well-being, quality of family relationship, quality of peer relationship, and total HRQoL score

Figure 5 displays the pooled ES (95% CI) estimates for the relationship between MF and HRQoL: 0.25 (0.12 to 0.37) for physical well-being, 0.11 (0.04 to 0.17) for psychological well-being, 0.08 (0.01 to 0.15) for quality of family relationship, 0.14 (0.03 to 0.25) for quality of peer relationship, and 0.09 (0.03 to 0.14) for total HRQoL score. Heterogeneity across the studies was rated as not important for psychological well-being and quality of family relationship (I^2^: 0% and 20.8%, respectively), moderate for quality of peer relationship and total HRQoL score (I^2^: 44.9% and 48.3%, respectively) and substantial for physical well-being (I^2^: 70.2%).

**Fig. 6:**
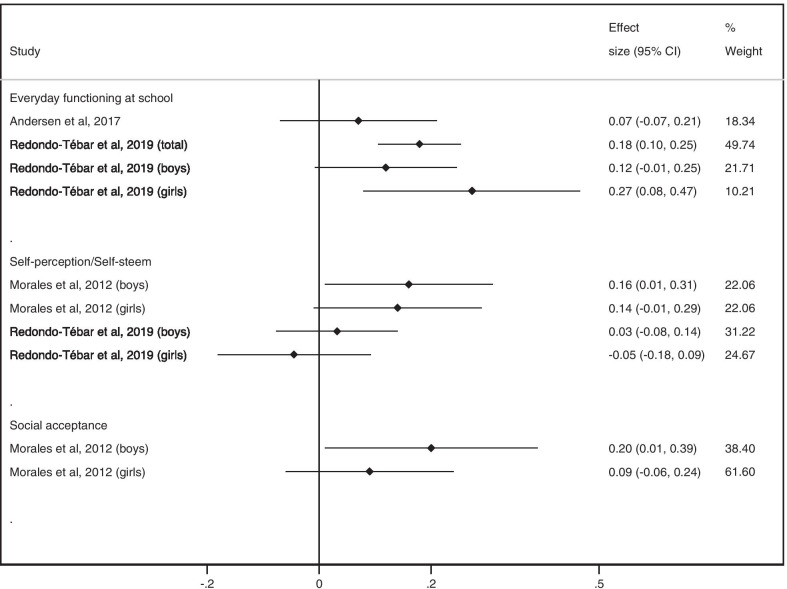
ES values of association between MF and everyday functioning at school, self-perception/self-esteem, and social acceptance

ES (95% CI) for the relationship between MF and everyday functioning at school ranged between 0.07 (− 0.07 to 0.21) and 0.27 (0.08 to 0.47). For self-perception, ES (95% CI) ranged between -0.05 (− 0.18 to 0.09) and 0.14 (− 0.01 to 0.29) in girls and between 0.03 (− 0.08 to 0.14) and 0.16 (0.01 to 0.31) in boys. For social acceptance, ES was 0.20 (95% CI 0.01 to 0.39) in boys. In girls, ES was 0.09 (95% CI − 0.06 to 0.24) (Fig. 6).

### Sensitivity analyses

Sensitivity analyses suggested that the pooled ES estimates did not change when studies were removed one by one (Additional file 1 and Additional file 2: Table S1 and Table S2).

### Publication bias

There was significant publication bias, as evidenced by both funnel plot asymmetry and Egger’s test for some outcome variables: for CRF, total HRQoL score (p = 0.020) and perceived health status (p = 0.007); and for MF, quality of family relationship (p = 0.090), total HRQoL score (p = 0.016), and physical well-being (p = 0.097) (Additional file 3 and Additional file 4: Table S3 and Table S4).

## Discussion

Fitness levels in childhood and adolescence are a lifelong marker of health and QoL. To our knowledge, this systematic review is the first synthesis on the relationship between physical fitness and HRQoL in the general population of healthy children and adolescents. The results of this meta-analysis suggest that there was a direct relationship between both CRF and MF and HRQoL mainly in physical, psychological and peer relationships; between CRF and school environment dimensions; and between MF and family relationships. Our estimates also show that CRF was not significantly associated with other dimensions of HRQoL such as self-perception/self-esteem, perceived health status or family relationships.

The available studies reported that good levels of physical fitness have a positive effect on physical, mental and social health in childhood and adolescence [7, 9, 46, 47]. In this regard, our meta-analysis shows that high levels of CRF and MF are associated with better HRQoL in both sexes, mainly affecting the domains of physical well-being, psychological well-being and quality of peer relationships.

Higher CRF and MF levels have been linked to better bone health and body composition [8, 43], lower risk of cardiometabolic diseases [9, 46] and greater muscular endurance [42], which may be related to improved perception of physical well-being identified in our meta-analysis. The positive association between CRF and MF and psychological health found in this meta-analysis could be explained by the effect of exercise on neuropeptides like serotonin and endorphins, which have a positive influence on mood [7]. Additionally, many children and adolescents engage in physical activity through sport or through play with friends of their age. Previous research has shown that friendship in young people can increase motivation to participate in physical activity and promote an increase in it [48]. Moreover, peer acceptance is of particular importance for youth development [49], directly affecting their perception of HRQoL.

Higher MF levels had a positive effect on family relationships, albeit with low ES. To our knowledge, no studies have analyzed the association between children's strength and relationships with parents, so it is difficult to compare our results with other studies. The existing literature has pointed out the importance of parents being active to ensure that children are active too [50] and the positive influence of parental exercise on children's fitness level [51, 52]. Thus, if children are active, they are more likely to have better fitness. Parental support for sport activities is positively associated with the enjoyment of sport and the importance it acquires in children's lives [53]. Arguably, active families are more involved in their children's physical activities and dedicate more time to active leisure, which could lead to a better parental relationship by spending time together on these activities.

The results of this review and meta-analysis also suggest a positive relationship between functioning at school (understood as the children’s perceptions of his/her cognitive capacity, concentration and learning, and their feelings about school), and CRF. These results can be compared with previous systematic reviews [54–56] in which the relationship between physical fitness and academic performance was evaluated, with better academic achievement in children with higher fitness levels observed in most of the studies included. In the same vein, one study that used functional magnetic resonance to evaluate brain activity in children in relation to fitness levels reported that children with higher physical fitness levels were better able to activate frontal and parietal brain regions, allowing them greater cognitive skills necessary for optimal functioning in school [57].

Finally, the results of our meta-analysis showed that the HRQoL domains related to self-perception, perceived health status and relationships with parents were not associated with CRF. These results could be explained by the fact we included studies with participants of different ages and that the variables within the domain groupings may have slightly different meanings.

Due to the subjective nature of QoL, it cannot be directly improved; however, it can be enhanced through increases in other behavioral factors. It is known that PA of different intensities, mainly vigorous [58, 59], can improve fitness in children and adolescents, and thus fitness could be a mediator between PA and HRQoL. In this regard, Eddolls et al. [44] found that enhancing CRF through increasing vigorous PA improved HRQoL in adolescents.

This systematic review has some potential limitations. First, although we have conducted and reported our review using existing guidelines [23], the characteristics and quality of the included studies could be a limitation. It should be noted that our findings come from observational studies; therefore, new research with experimental designs might reinforce the existing evidence. Second, three of the 11 studies included were based on parent-reported rather than child-reported HRQoL questionnaires, and considering that previous studies have shown discrepancies between parent and child scores on proxy questionnaires [60, 61], the results should be interpreted with caution. Third, it should be borne in mind when interpreting the results that this meta-analysis includes studies that have used different HRQoL questionnaires, so it is possible that the variables in the domain groupings do not refer to exactly the same constructs, even if they share similar meanings. Fourth, the studies included in this systematic review were conducted only in European population, which may be a limitation when generalizing the results obtained. Fifth, an analysis of the effects of fitness on HRQoL by age group could not be performed because only one study reported their results separately for children and adolescents. Finally, gray literature was not included in the search and we only included studies published in English or Spanish. This may result in a loss of information for the results reported.

## Conclusions

The results of this systematic review show that there is consistent evidence to support that healthy children and adolescents with high CRF and MF levels have better HRQoL than those peers that do not, showing greater physical and psychological well-being and better quality peer relationships. The findings of this systematic review could be of interest to researchers, policy makers, and practitioners in the areas of physical activity, education and health care, providing the basis for the development of effective action plans to promote physical activity, including programs that reinforce the role of CRF and MF. These programs could include projects for sustainable mobility and active commuting to and from school, modifications to the schoolyard environment to facilitate strength and endurance games (e.g., with fixed elements such as bars for hanging, tire wheels for pushing and moving or climbing walls, or mobile elements such as skipping ropes, balls, etc.), as well as organized physical activities to improve fitness during recess. Games of strength and endurance should be enhanced and reinforced during physical education hours and implemented in children’s and adolescents’ daily leisure time, which will have positive effects on their QoL and increase their physical, psychological, and social well-being.

## Supplementary Information

**Additional file 1. Supplementary Table 1**: Sensitivity analysis for CRF and HRQoL.

**Additional file 2. Supplementary Table 2**: Sensitivity analysis for MF and HRQoL.

**Additional file 3: Supplementary Table 3**: . Publication bias for CRF.

**Additional file 4. Supplementary Table 4**: Publication bias for MF.

## Data Availability

Not applicable.

## Abbreviations

CRF: Cardiorespiratory fitness
MF: Muscular fitness
HRQoL: Health-related quality of life
QoL: Quality of life
ES: Effect size
PRISMA: Preferred Reporting Items for Systematic Reviews and Meta-Analyses
STROBE: Strengthening the Reporting of Observational studies in Epidemiology
EPHPP: Effective Public Health Practice Project
CI: Confidence intervals
EQ-5D: EuroQol Group 5-dimension questionnaire
VAS: Visual Analog Scale
HBSC: Health Behavior in School-aged Children
PedsQL: Pediatric Quality of Life Inventory
CHIP-CE: Child Health and Illness Profile
IFIS: International Fitness Scale
