# Supplementary material for: Relationship between both cardiorespiratory and muscular fitness and health-related quality of life in children and adolescents: a systematic review and meta-analysis of observational studies
Source: Health Qual Life Outcomes. 2021 Apr 21;19:127. doi: 10.1186/s12955-021-01766-0 (PMC8059195; doi:10.1186/s12955-021-01766-0)
Supplement: Supplementary file 1 — Additional file 1. Supplementary Table 1: Sensitivity analysis for CRF and HRQoL. [file 12955_2021_1766_MOESM1_ESM.docx]

**Supplementary Table 1*.*** Sensitivity analysis for CRF and HRQoL

|  | **es** | **ll** | **ul** |
| --- | --- | --- | --- |
| **Physical well-being** |  |  |  |
| Andersen et al, 2017 | 0.205 | 0.100 | 0.309 |
| Borras et al, 2011 | 0.201 | 0.108 | 0.294 |
| Eddolls et al, 2018 | 0.210 | 0.119 | 0.300 |
| Gerber et al, 2011 | 0.188 | 0.092 | 0.284 |
| Morales et al, 2012 (boys) | 0.141 | 0.086 | 0.196 |
| Morales et al, 2012 (girls) | 0.179 | 0.089 | 0.268 |
| Palou et al, 2012 | 0.182 | 0.088 | 0.276 |
| Redondo-Tébar et al, 2019 (boys) | 0.194 | 0.088 | 0.299 |
| Redondo-Tébar et al, 2019 (girls) | 0.194 | 0.097 | 0.290 |
| **Psychological well-being** |  |  |  |
| Andersen et al, 2017 | 0.1435 | 0.0401 | 0.2469 |
| Borras et al, 2011 | 0.1650 | 0.0335 | 0.2964 |
| Gerber et al, 2011 | 0.1936 | 0.0495 | 0.3377 |
| Morales et al, 2012 (boys) | 0.2075 | 0.0604 | 0.3546 |
| Morales et al, 2012 (girls) | 0.1845 | 0.0490 | 0.3199 |
| Palou et al, 2012 | 0.1973 | 0.0560 | 0.3387 |
| Redondo-Tébar et al, 2019 (boys) | 0.2316 | 0.1196 | 0.3436 |
| Redondo-Tébar et al, 2019 (girls) | 0.2032 | 0.0669 | 0.3396 |
| **Perceived health status** |  |  |  |
| Borras et al, 2011 | 0.0853 | -0.2092 | 0.3799 |
| Lämmle et al, 2013 | 0.3084 | 0.0795 | 0.5374 |
| Padilla-Moledo et al, 2011 (adolescents) | 0.1868 | -0.2054 | 0.5791 |
| Padilla-Moledo et al, 2011 (children) | 0.2112 | -0.2011 | 0.6236 |
| Palou et al, 2012 | 0.2229 | -0.1866 | 0.6323 |
| Saavedra et al, 2013 | 0.2050 | -0.2023 | 0.6124 |
| **Quality of family relationship** |  |  |  |
| Andersen et al, 2017 | 0.0107 | -0.0595 | 0.0809 |
| Gerber et al, 2016 | 0.0959 | -0.0277 | 0.2196 |
| Morales et al, 2012 (boys) | 0.0637 | -0.0783 | 0.2058 |
| Morales et al, 2012 (girls) | 0.0698 | -0.0651 | 0.2047 |
| Padilla-Moledo et al, 2011 (adolescents) | 0.0685 | -0.0653 | 0.2023 |
| Padilla-Moledo et al, 2011 (children) | 0.0608 | -0.0740 | 0.1957 |
| Redondo-Tébar et al, 2019 (boys) | 0.0929 | -0.0320 | 0.2179 |
| Redondo-Tébar et al, 2019 (girls) | 0.0682 | -0.0690 | 0.2054 |
| **Quality of peer relationship** |  |  |  |
| Andersen et al, 2017 | 0.1182 | 0.0029 | 0.2334 |
| Gerber et al, 2016 | 0.1401 | 0.0176 | 0.2627 |
| Morales et al, 2012 (boys) | 0.1145 | 0.0083 | 0.2207 |
| Morales et al, 2012 (girls) | 0.1348 | 0.0129 | 0.2568 |
| Padilla-Moledo et al, 2011 (adolescents) | 0.1578 | 0.0428 | 0.2729 |
| Padilla-Moledo et al, 2011 (children) | 0.1870 | 0.1022 | 0.2718 |
| Redondo-Tébar et al, 2019 (boys) | 0.1478 | 0.0226 | 0.2730 |
| Redondo-Tébar et al, 2019 (girls) | 0.1492 | 0.0276 | 0.2707 |
| **HRQoL** |  |  |  |
| Evaristo et al, 2019 | 0.1863 | 0.0999 | 0.2726 |
| Gerber et al, 2016 | 0.2067 | 0.1171 | 0.2962 |
| Gálvez et al, 2015 (boys) | 0.1621 | 0.1095 | 0.2147 |
| Gálvez et al, 2015 (girls) | 0.1880 | 0.1073 | 0.2687 |
| Marques et al, 2017 (boys) | 0.2285 | 0.1243 | 0.3328 |
| Marques et al, 2017 (girls) | 0.2297 | 0.1257 | 0.3338 |
| Morales et al, 2012 (boys) | 0.2140 | 0.1218 | 0.3061 |
| Morales et al, 2012 (girls) | 0.1958 | 0.1090 | 0.2826 |
| Redondo-Tébar et al, 2019 (boys) | 0.2224 | 0.1283 | 0.3165 |
| Redondo-Tébar et al, 2019 (girls) | 0.2059 | 0.1191 | 0.2927 |

es: effect size; ll: lower limit; ul: upper limit
