# Supplementary material for: Relationship between both cardiorespiratory and muscular fitness and health-related quality of life in children and adolescents: a systematic review and meta-analysis of observational studies
Source: Health Qual Life Outcomes. 2021 Apr 21;19:127. doi: 10.1186/s12955-021-01766-0 (PMC8059195; doi:10.1186/s12955-021-01766-0)
Supplement: Supplementary file 2 — Additional file 2. Supplementary Table 2: Sensitivity analysis for MF and HRQoL. [file 12955_2021_1766_MOESM2_ESM.docx]

**Supplementary Table 2*.*** Sensitivity analysis for MF and HRQoL

|  | **es** | **ll** | **ul** |
| --- | --- | --- | --- |
| **Physical well-being** |  |  |  |
| Andersen et al, 2017 | 0.2865 | 0.1107 | 0.4623 |
| Morales et al, 2012 (boys) | 0.1917 | 0.0945 | 0.2890 |
| Morales et al, 2012 (girls) | 0.2229 | 0.0957 | 0.3500 |
| Redondo-Tébar et al, 2019 (boys) | 0.2583 | 0.0927 | 0.4239 |
| Redondo-Tébar et al, 2019 (girls) | 0.2953 | 0.1586 | 0.4319 |
| **Psychological well-being** |  |  |  |
| Andersen et al, 2017 | 0.0942 | 0.0211 | 0.1673 |
| Morales et al, 2012 (boys) | 0.1096 | 0.0383 | 0.1809 |
| Morales et al, 2012 (girls) | 0.1117 | 0.0404 | 0.1831 |
| Redondo-Tébar et al, 2019 (boys) | 0.1135 | 0.0452 | 0.1819 |
| Redondo-Tébar et al, 2019 (girls) | 0.0992 | 0.0198 | 0.1787 |
| **Quality of family relationship** |  |  |  |
| Andersen et al, 2017 | 0.0728 | -0.0040 | 0.1496 |
| Morales et al, 2012 (boys) | 0.0767 | -0.0106 | 0.1640 |
| Morales et al, 2012 (girls) | 0.0528 | -0.0140 | 0.1195 |
| Redondo-Tébar et al, 2019 (boys) | 0.1033 | 0.0110 | 0.1966 |
| Redondo-Tébar et al, 2019 (girls) | 0.1040 | 0.0286 | 0.1795 |
| **Quality of peer relationship** |  |  |  |
| Andersen et al, 2017 | 0.1478 | 0.0198 | 0.2758 |
| Morales et al, 2012 (boys) | 0.1018 | 0.0226 | 0.1810 |
| Morales et al, 2012 (girls) | 0.1326 | -0.0085 | 0.2737 |
| Redondo-Tébar et al, 2019 (boys) | 0.1800 | 0.0613 | 0.2987 |
| Redondo-Tébar et al, 2019 (girls) | 0.1680 | 0.0152 | 0.3208 |
| **HRQoL** |  |  |  |
| Evaristo et al, 2019 | 0.0599 | 0.0139 | 0.1059 |
| Marques et al, 2017 (boys) | 0.1070 | 0.0416 | 0.1724 |
| Marques et al, 2017 (girls) | 0.1072 | 0.0434 | 0.1710 |
| Morales et al, 2012 (boys) | 0.0782 | 0.0223 | 0.1341 |
| Morales et al, 2012 (girls) | 0.0898 | 0.0270 | 0.1526 |
| Redondo-Tébar et al, 2019 (boys) | 0.0850 | 0.0227 | 0.1473 |
| Redondo-Tébar et al, 2019 (girls) | 0.0866 | 0.0224 | 0.1507 |

es: effect size; ll: lower limit; ul: upper limit
