# Supplementary material for: Relationship between both cardiorespiratory and muscular fitness and health-related quality of life in children and adolescents: a systematic review and meta-analysis of observational studies
Source: Health Qual Life Outcomes. 2021 Apr 21;19:127. doi: 10.1186/s12955-021-01766-0 (PMC8059195; doi:10.1186/s12955-021-01766-0)
Supplement: Supplementary file 3 — Additional file 3: Supplementary Table 3: . Publication bias for CRF. [file 12955_2021_1766_MOESM3_ESM.docx]

**Supplementary Table 3.** Publication bias for CRF

| **Dimension** | **Coef.** | **ll** | **ul** | **p** |
| --- | --- | --- | --- | --- |
| Physical well-being | 1.4049 | -1.6354 | 4.4452 | 0.311 |
| Psychological well-being | 0.8412 | -4.3824 | 6.0648 | 0.707 |
| Perceived health status | 7.3935 | 3.3381 | 11.4489 | 0.007 |
| Self-perception/Self-esteem | 3.4967 | -8.1145 | 15.1079 | 0.409 |
| Quality of family relationship | -1.2687 | -8.5218 | 5.9845 | 0.684 |
| Quality of peer relationship | -4.0879 | 10.6995 | -2.5238 | 0.181 |
| Everyday functioning at school | -2.2861 | -10.3615 | 5.7893 | 0.476 |
| HRQoL | 2.2528 | 0.4653 | 4.0402 | 0.020 |

ll: lower limit; ul: upper limit
