# Supplementary material for: Relationship between both cardiorespiratory and muscular fitness and health-related quality of life in children and adolescents: a systematic review and meta-analysis of observational studies
Source: Health Qual Life Outcomes. 2021 Apr 21;19:127. doi: 10.1186/s12955-021-01766-0 (PMC8059195; doi:10.1186/s12955-021-01766-0)
Supplement: Supplementary file 4 — Additional file 4. Supplementary Table 4: Publication bias for MF. [file 12955_2021_1766_MOESM4_ESM.docx]

**Supplementary Table 4.** Publication bias for MF

| **Dimension** | **Coef.** | **ll** | **ul** | **p** |
| --- | --- | --- | --- | --- |
| Physical well-being | 3.8012 | -1.2735 | 8.8760 | 0.097 |
| Psychological well-being | -1.7379 | -4.6479 | 1.1720 | 0.154 |
| Quality of family relationship | 3.2128 | -0.9297 | 7.3553 | 0.090 |
| Quality of peer relationship | 2.4407 | -3.4790 | 8.3604 | 0.281 |
| HRQoL | 3.0405 | .85528 | 5.2257 | 0.016 |

ll: lower limit; ul: upper limit
